# The Dipeptidyl Peptidase Family, Prolyl Oligopeptidase, and Prolyl Carboxypeptidase in the Immune System and Inflammatory Disease, Including Atherosclerosis

**DOI:** 10.3389/fimmu.2015.00387

**Published:** 2015-08-07

**Authors:** Yannick Waumans, Lesley Baerts, Kaat Kehoe, Anne-Marie Lambeir, Ingrid De Meester

**Affiliations:** ^1^Laboratory of Medical Biochemistry, Department of Pharmaceutical Sciences, University of Antwerp, Antwerp, Belgium

**Keywords:** dipeptidyl peptidase, prolyl oligopeptidase, fibroblast activation protein α, prolyl carboxypeptidase, inflammation, immunophysiology, atherosclerosis

## Abstract

Research from over the past 20 years has implicated dipeptidyl peptidase (DPP) IV and its family members in many processes and different pathologies of the immune system. Most research has been focused on either DPPIV or just a few of its family members. It is, however, essential to consider the entire DPP family when discussing any one of its members. There is a substantial overlap between family members in their substrate specificity, inhibitors, and functions. In this review, we provide a comprehensive discussion on the role of prolyl-specific peptidases DPPIV, FAP, DPP8, DPP9, dipeptidyl peptidase II, prolyl carboxypeptidase, and prolyl oligopeptidase in the immune system and its diseases. We highlight possible therapeutic targets for the prevention and treatment of atherosclerosis, a condition that lies at the frontier between inflammation and cardiovascular disease.

## Introduction

**Figure 1:**
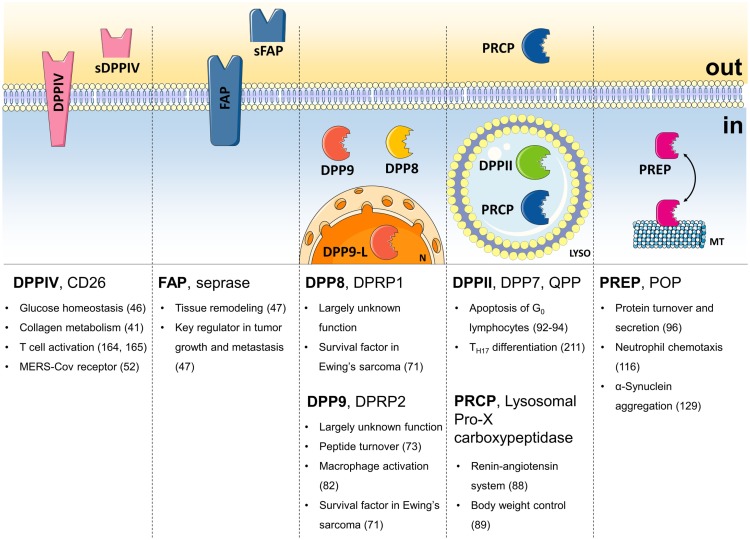
**The DPP family**. Summary of the names, the localization, and most important field of action of all members of the DPP family. sDPPIV, soluble DPPIV; sFAP, soluble FAP; DPP9-L, long form of DPP9; N, nucleus; LYSO, lysosome; MT, microtubules; in, intracellular; out, extracellular.

Research from over the past 20 years has implicated the dipeptidyl peptidase (DPP) family in various physiological processes and pathologies of the immune system. Usually only four prolyl-specific peptidases are considered: DPPIV (EC 3.4.14.5), fibroblast activation protein α (FAP; EC 3.4.21.B28), and the more recently discovered DPP8 and DPP9 (EC 3.4.14). However, due to similarities in substrate specificity and structural homology, it is more relevant to consider a broader family that also includes prolyl oligopeptidase (PREP; EC 3.4.21.26), dipeptidyl peptidase II (DPPII) (EC 3.4.14.2), and prolyl carboxypeptidase (PRCP; EC 3.4.16.2). First, DPPII and PRCP share the α/β hydrolase fold with the other DPPs and the catalytic triad is completely conserved in both enzymes (2). Moreover, DPPII can cleave several DPPIV substrates *in vitro* (3). Conversely, due to its substrate preference for tripeptides (4), DPPII could actually be considered as a prolyl carboxytripeptidase, emphasizing its similarities to PRCP. Another argument for considering a broader family stems from the fact that functional studies on the role of peptidases rely heavily on the use of enzyme inhibitors and many of the inhibitors used in earlier studies are now known to inhibit more than one family member. For example, early studies on DPPIV used inhibitors which we now know also inhibit DPPII, DPP8, DPP9, FAP, and/or PREP due to their sequential and/or structural similarity [e.g., Ref. (5–9)]. PRCP is known to be inhibited by KYP-2047 and Z-Pro-Prolinal at higher concentrations, which have often been used for the functional study of PREP [e.g., Ref. (10–12)]. Table 1 summarizes the most commonly used DPP inhibitors and their selectivity compared to DPPIV. In view of the aforementioned reasons and for the sake of simplicity, we will use “DPP family” as a blanket term, which includes DPPII, PRCP, and PREP even though strictly speaking they are not DPPs. Figure 1 provides a general overview of this broadly defined DPP family. The roles of various family members in certain aspects of the immune system or immune dysfunction have been reviewed in the past [e.g., Ref. (13–15)]. In this review, we provide a comprehensive discussion and update on the roles of DPPIV, DPPII, DPP8, DPP9, FAP, PREP, and PRCP in the immune system and inflammatory disease. We highlight the role of these enzymes in atherosclerosis, a condition that lies at the frontier between inflammation and cardiovascular disease, as the DPP family encompasses possible therapeutic targets for the prevention and treatment of this disease.

**Table 1:** **Overview of commonly used inhibitors within the DPP family and the ratio of inhibitor needed to inhibit the respective DPP family member compared to what is needed to inhibit DPPIV**.

Inhibitors		DPPII	DPP8	DPP9	FAP	PREP	PRCP	Reference
Clinical	*Alogliptin*	>14,000	>14,000	>14,000	>14,000	>14,000	ND	(16)
	*Linagliptin*	>100,000	40,000	>10,000	89	>100,000	ND	(17)
	*Saxagliptin*	>50,000	390	77	>4,000	ND	ND	(18)
	*Sitagliptin*	>5,550	>5,550	>2,660	>5,550	>5,550	ND	(19)
	*Talabostat*	4	8	4	3	44	ND	
	*Vildagliptin*	>100,000	270	32	285	60,000	ND	(20, 21)
Experimental	*1G244*	1	<10^−3^	<10^−3^	1	ND	ND	(21)
	*KYP2047*	1	ND	1	1	<10^−4^	1	(12, 22)
	*UAMC01110*	1	ND	0.5	<10^−4^	0.1	ND	(23)
	*UAMC00039*	<10^−5^	1	2	>10	ND	ND	(24, 25)
	*Z-Pro-Prolinal*	ND	ND	ND	ND	<10^−2^	*	(26, 27)

*Partly adapted from Deacon (28)*.

*Z-Pro-Prolinal is currently used as an inhibitor for PRCP although it is *in vitro* 10^3^ times more selective toward PREP.

## A Brief Guide to the DPP Family

### Dipeptidyl peptidase IV

The prototypical DPP, DPPIV (often DPP4 in medical jargon) cleaves off an N-terminal dipeptide from peptides with Pro or Ala on the penultimate position. Its localization as a soluble enzyme in body fluids, or anchored in the plasma membrane of cells provides it with the necessary access to cleave a wide range of bioactive peptides. As such, it can modify their biological activity. Glucagon-like peptide (GLP)-1 and -2, and glucose-dependent insulinotropic peptide (GIP) (29, 30), substance P (31), neuropeptide Y (NPY) (32), stromal cell-derived factor-1α/β (SDF-1α/β or CXCL12) (33), granulocyte macrophage colony-stimulating factor (GM-CSF) (1), CXCL10 (34–36), and high-mobility group box 1 (HMGB1) (37) have been identified as physiological substrates, while others, such as RANTES, have been proposed based on *in vitro* experiments [e.g., Ref. (38)]. DPPIV also performs many of its physiological functions through interactions with other proteins, such as collagen, fibronectin, adenosine deaminase (ADA), caveolin-1, and the mannose-6-phosphate/insulin-like growth factor II receptor (M6P/IGFIIR) (39–41). Some of those will be discussed in more detail below.

Dipeptidyl peptidase IV is well known for its role in glucose homeostasis. It has become a validated therapeutic target for the treatment of type 2 diabetes (T2D) (46). DPPIV inhibitors reduce the rate of GLP-1 inactivation (Boxes 1 and 2). It has also been shown to be involved in cancer biology. The role of the DPP family in cancer has been addressed in several other reviews (39, 47–51). Finally, DPPIV has recently come back into the center of attention as the receptor for the MERS coronavirus (52).

Box 1 **Incretins.**

The incretins are a group of glucose-lowering molecules produced by the intestines. The best known incretin is glucagon-like peptide-1 (GLP-1). This incretin is derived from proglucagon and secreted after a meal from L-cells in the distal ileum and colon. In the pancreas, it induces insulin secretion and biosynthesis while lowering glucagon secretion. In addition, GLP-1 increases the β-cell mass, thereby restoring insulin production. It is clear that GLP-1 also has functions outside glucose metabolism. Its receptor, GLP-1-R, is not only found in the pancreas but also expressed in brain, lung, kidney, stomach, and heart (42, 43). Recently, it was shown that stimulation after myocardial infarction reduces the infarct size (44, 45). Currently, GLP-1 agonists are approved for the treatment of type 2 diabetes. These incretin mimetics seem to have a slightly better efficacy as DPPIV inhibitors and lead more frequently to weight loss. Unfortunately, an important drawback for their therapeutic use is that they can only be administered by subcutaneous injection (46).

Box 2 **DPPIV inhibitors.**

Dipeptidyl peptidase IV inhibitors prolong the biological half-life of the incretins and are therefore used for the treatment of type 2 diabetes. Sitagliptin, vildagliptin, saxagliptin, linagliptin, and alogliptin are DPPIV inhibitors currently available on the market for treatment of type 2 diabetes. Sitagliptin and alogliptin are highly selective toward DPPIV *in vitro*, whereas vildagliptin and saxagliptin are less selective with regard to DPP8 and 9, and linagliptin with regard to FAP (28). Their clinical efficacy and safety in the use of type 2 diabetes seem comparable as far as can be judged from the data available.

There is a growing interest toward a use outside type 2 diabetes as it has become clear that DPPIV inhibitors have pleiotropic effects. While negative effects have been found in heart failure (53), some studies suggest them as a possible therapeutic strategy in cardiovascular pathologies (28, 54). The SITAGRAMI trial and follow-up studies revealed that the combination of a DPPIV inhibitor with granulocyte-colony-stimulating factor or in monotherapy presents a therapeutic option after myocardial infarction (55, 56). As stated above, the mechanism is not yet clear but may be explained by a longer biological half-life of DPPIV substrates, glucagon-like peptide-1, B-type natriuretic peptide, and stromal cell-derived factor-1 α/β. All three peptides have a cardioprotective effect that is abolished by DPPIV-mediated cleavage. For an extensive review of the involved substrates, see Matheeussen et al. (43).

### Fibroblast activation protein α

Fibroblast activation protein α, also known as seprase can present itself as a type II transmembrane protein or as a shedded plasma protease (57). In the latter case, it is also known as antiplasmin-cleaving enzyme, which converts α2-antiplasmin into a more active form, suppressing fibrinolysis (58). Some of the known DPPIV substrates were later found to be cleaved *in vitro* by FAP as well (59), though any physiological relevance remains unclear.

Unlike DPPIV, FAP also possesses a gelatinase activity. This enables FAP to degrade proteins of the extracellular matrix (60). This is of particular interest with regard to its involvement in a number of pathological processes (47). FAP is highly induced during inflammation, activation of hepatic stellate cells in liver cirrhosis and strongly expressed by mesenchymal cells of remodeling tissue (47, 61). FAP is also a key regulator during tumor growth and metastasis (47). As all these processes require degradation of the extracellular matrix, FAP’s involvement in these pathologies is most likely associated with its gelatinase activity (51). Its role in cancer biology has been reviewed before (47, 62). It is interesting to note that, so far, in clinical trials Talabostat has shown minimal or no clinical benefit for the treatment of metastatic colorectal cancer, advanced non-small cell lung cancer, or stage IV melanoma (63–65). It should be mentioned, however, that Talabostat is a broad-range inhibitor also targeting DPPIV, DPP8, and DPP9.

### Dipeptidyl peptidases 8 and 9

Dipeptidyl peptidases 8 and DPP9 show DPPIV-like activity and share a very high-sequence similarity to each other (77% aa similarity, 57% aa identity) (24). These cytoplasmic enzymes have several isoforms. It has been a matter of debate whether all are expressed as protein in cells and, if so, whether they are active (66–69). Interestingly, the N-terminal extension of the longer DPP9 variant contains a nuclear localization signal and, indeed, this form localizes to the nucleus (69). DPP8 has been shown to cleave a number of DPPIV chemokine substrates *in vitro* (70). Another DPPIV substrate, NPY, has indirectly been shown to be a DPP8 and DPP9 substrate as well (71). Efforts have been made to find intracellular DPP8 and 9 substrates using a peptidomic approach (72), but so far it has been hard to attribute physiological relevance to the possible substrates beyond the role of DPP8 and 9 in intracellular peptide turnover (73).

The physiological functions of DPP8 and DPP9 are still not properly understood. Mainly, a lack of available knockout animals, specific inhibitors, and substrates has hampered progress (24). A mouse model has been established with a targeted inactivation of DPP9 enzymatic activity (74), but homozygous DPP9-inactive neonates die within 8–24 h after birth. Despite these limitations, some indications toward their role are surfacing. Using immunohistochemistry, DPP8 and 9 were found associated with spermatozoids and spermatids and the short mRNA of DPP8 is predominantly expressed in testes (75, 76), suggesting a role in spermatogenesis and male fertility. Recent work has found SUMO1 to be an allosteric activator of DPP9 (77), whereas a small peptide corresponding to the interaction surface of SUMO1 is a non-competitive inhibitor of DPP8 and DPP9 (78). A genome-wide association study has linked DPP9 to idiopathic pulmonary fibrosis (79).

Finally, a number of studies have shown a role for DPP8 and DPP9 in apoptosis (71, 80–83). Two studies showed that overexpression enhanced induced apoptosis and impaired cell adhesion and migration (80, 81). Conversely, DPP8/9 inhibition in tumor cells decreased the number of viable cells because of a decreased cleavage of pro-apoptotic NPY (71). In macrophages, inhibition caused a marginal, yet significant increase in apoptosis, independent of NPY cleavage (82). Interestingly, vildagliptin, a DPPIV inhibitor already on the market to treat type 2 diabetes, but with poorer selectivity toward DPP8 and 9, was shown to enhance parthenolide’s anti-leukemic activity through its inhibition of DPP8 and 9, and not DPPIV (83).

### Dipeptidyl peptidase II and prolyl carboxypeptidase

Prolyl carboxypeptidase, also called angiotensinase C or lysosomal Pro-X carboxypeptidase, is a lysosomal carboxypeptidase sharing strong sequence homology with the likewise lysosomal DPPII (4, 84). PRCP preferentially cleaves off the C-terminal amino acid when Ala or Pro is in the penultimate position, while DPPII targets N-terminal X-Pro or X-Ala dipeptides (85, 86). In addition to a structural similarity, PRCP and DPPII have partially overlapping substrate specificities due to DPPII’s preference for tripeptide substrates (4). Perhaps surprisingly, Gly-Pro-pNA and Ala-Pro-pNA, two typical synthetic DPP substrates, have actually been used to perform PRCP activity measurements (87).

Prolyl carboxypeptidase is particularly known as one of the key enzymes of the renin–angiotensin system (RAS). It inactivates the vasoactive peptides angiotensin II and angiotensin III by cleaving off the C-terminal Phe (88). α-Melanocyt-stimulating hormone 1–13, an anorexigenic neuromodulator, is inactivated by PRCP, implying a role in body weight control (89). Based on the involvement of PRCP in the conversion of these peptide hormones, the enzyme has also been associated with diseases, such as hypertension, diabetes mellitus, obesity, inflammation, and cardiovascular dysfunction (90, 91).

Dipeptidyl peptidase II has no known natural substrates. The DPPIV substrate substance P has been shown to be cleaved by DPPII *in vitro* (3), but much less efficiently, casting doubt over any physiological relevance. It has been shown that inhibition or silencing of DPPII causes apoptosis of quiescent *G*_0_ lymphocytes (92–94). On the other hand, a highly specific DPPII inhibitor, UAMC00039, did not induce apoptosis, autophagy, or necrosis in human leukocytes (25, 95), but this study did not specifically look at quiescent cells or lymphocytes. Finally, changes in DPPII activity levels have been observed in a number of pathologies, such as neurodegenerative disorders, myopathies, cancer, and gastro-intestinal disorders (4).

### Prolyl oligopeptidase

Prolyl oligopeptidase is an oligopeptidase with endopeptidase activity. It has been shown to be localized in the cytoplasm (96–99), but given its ability to inactivate several neuropeptides *in vitro* by limited proteolysis (100–115), its involvement in the *in vivo* generation of immunoactive peptides *N*-acetyl-prolyl-glycyl-proline and *N*-acetyl-seryl-aspartyl-lysyl-proline (116, 117), and its presence in plasma (118, 119), it most likely also has an extracellular role.

Initial interest for PREP derived from the positive effects of PREP inhibitors on scopolamine-induced amnesia in rats (120–123). PREP inhibition was also found to promote neuronal survival and neurite outgrowth of cerebellar granule cells (124). However, a recent study in mice shows that the lack of PREP *in vivo* causes a reduction of synaptic spine density in the hippocampal region along with reduced long-term potentiation and memory functions (125).

Many of PREP’s functions are mediated through its interactions with other proteins. PREP is known to interact with GAP-43 (126, 127), α-tubulin (96), and GADPH (128). Its most studied interaction is with α-synuclein (126), reviewed in Ref. (129). PREP and α-synuclein have been shown to co-localize in cell models of stress and in the substantia nigra of post-mortem Parkinson’s disease brain (11, 130). *In vitro*, the aggregation rate of α-synuclein increases in the presence of high concentrations of PREP, which is abolished through active site inhibitors of PREP and absent with a catalytically impaired PREP mutant (131). *In vivo*, PREP inhibition reduces α-synuclein aggregates in a cellular and animal model for Parkinson’s disease (11).

## The DPP Family in the Immune System

### The DPP family in the innate immune system

#### DPP Family Members in Monocytes and Macrophages

**Figure 2:**
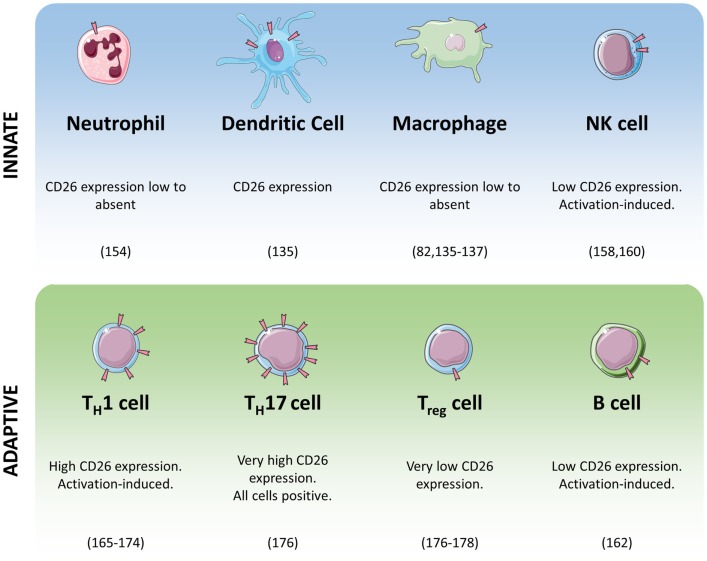
**Summary of CD26/DPPIV expression in cells of the immune system**.

The role of DPPIV in monocytes and macrophages has been somewhat contested. Whereas DPPIV’s presence on monocytes and macrophages has been shown repeatedly in mice and rats (132–134), its expression in human monocytes and macrophages is less obvious. Figure 2 shows an overview of the expression of DPPIV throughout the immune system. In visceral obesity, DPPIV expression is low on peripheral blood monocytes, macrophages, and dendritic cells, but it is upregulated *in vitro* after differentiation and activation of isolated monocytes into macrophages or dendritic cells, and *in vivo* locally in adipose tissue (135). Interestingly, the authors showed that macrophage- or dendritic cell-associated DPPIV most likely binds ADA, promoting local degradation of adenosine, a T-cell proliferation suppressor, thereby inducing T-cell proliferation (135). Three other studies also found no to low DPPIV expression or activity associated with human monocytes and/or macrophages (82, 136–138). Others have investigated DPPIV in monocyte- or macrophage-like cell lines (136, 137, 139–144). In HL-60 cells, its expression has been found to be regulated by differentiation into macrophage-like cells (139). DPPIV inhibitor alogliptin can affect ERK activation, MMP1 and IL-6 secretion in U937 cells (140, 141). However, these studies employed alogliptin at concentrations lower than its IC_50_ for DPPIV. It is therefore questionable whether the observed effects were mediated by DPPIV at all. On the other hand, proliferation is reduced in the presence of a DPP inhibitor in U937 cells expressing high levels of DPPIV, but not in the same cell type expressing low levels of DPPIV (144). Moreover, the same inhibitor causes the former cells to secrete lower amounts of IL-1β, but higher amounts of TNFα (144). It could be that inhibition merely increases TNFα’s half-life, as DPPIV has been implicated in its degradation in U937 cells (137). In THP-1 cells, DPPIV inhibitors alogliptin and sitagliptin both reduced these cells’ chemotactic potential (142). DPPIV inhibitors sitagliptin and NVPDPP728 also reduced NLRP3, TLR4, and IL-1β expression and increased GLP-1R expression in THP-1 cells and this effect was blocked through PMA differentiation (143). Importantly, such cell lines have been derived from different types of myeloid leukemia, and as it is known that DPPIV expression is often dysregulated in cancer (47–51), the physiological relevance of these findings remains uncertain. FAP has been shown on tumor-associated macrophages in human breast cancer (145).

Dipeptidyl peptidase 8/9 activity has been found in human monocytes and U937 cells (136). DPP8 was found associated with activated microglia/macrophages in a rat model of cerebral ischemia (146). DPP8 and 9 are abundantly present in macrophage-rich regions of atherosclerotic plaques (82). Interestingly, DPP9 is upregulated after *in vitro* monocyte-to-macrophage differentiation. Moreover, inhibition or RNA silencing of DPP9 attenuates pro-inflammatory M1, but not M2, macrophage activation (82).

In rats, DPPII is expressed in tissue-resident macrophages (147, 148). Humans show DPPII activity in monocytes as well as U937 cells (25, 136). Human blood derived alveolar macrophages show high-PRCP activity (138, 149). Interestingly, in a mouse *in vivo* angiogenesis assay, macrophage infiltration into the wound was increased in mice with a PRCP deletion (150).

Prolyl oligopeptidase activity has been shown in mouse and rat peritoneal macrophages and in rat pulmonary macrophages (134, 151, 152). Its activity in mouse peritoneal macrophages is increased after thioglycollate ellicitation (134). In addition, PREP has been identified as a neurotoxic component in the supernatant of activated THP-1 cells, which are monocyte-like cells (153). Apparently, these cells secrete PREP upon activation with IFNγ and LPS and partly because of this, their supernatant is toxic to neuroblastoma SH-SY5Y cells, as shown through the use of PREP-specific inhibitors (153). PREP’s mode of action in this remains unclear.

#### DPP Family Members in Granulocytes

Recently, a study showed that DPPIV acts as a chemorepellent for human and murine neutrophils (154). Adding recombinant DPPIV to purified human neutrophils in an Insall chamber causes the neutrophils to migrate away from the higher concentration of DPPIV. This effect is blocked by DPPIV inhibitors, meaning that the effect is mediated through DPPIV’s enzymatic activity, although a candidate substrate is not obvious. Moreover, in a mouse model of acute respiratory distress syndrome, oropharyngeal aspiration of DPPIV prevented accumulation of neutrophils in the lung (154). By contrast, PREP is involved in the generation of prolyl-glycyl-proline, a collagen fragment that is an efficient neutrophil chemoattractant (155). Human peripheral blood neutrophils contain PREP activity and are themselves capable of generating prolyl-glycyl-proline after LPS-activation, alluding to a self-sustaining pathway of neutrophil inflammation (116). PRCP is also abundantly expressed in human neutrophils (90).

The recruitment of eosinophils is affected by DPPIV activity. CCL11, also known as eotaxin, is a DPPIV substrate and cleavage by DPPIV prevents the activation of its receptor CCR3 (156). In rats, it was shown that administration of CCL11 results in eosinophil recruitment and this recruitment is significantly more effective in DPPIV-deficient F344 mutants (156).

Finally, DPPII activity has been reported in the granules of mast cells in several publications (147, 148, 157). It is released from peritoneal mast cells upon degranulation and is apparently inhibited by histamine and Zn^2+^ at concentrations present in the granules of mast cells (157).

#### DPP Family Members in Natural Killer Cells

Dipeptidyl peptidase 4 is present in low amounts on freshly isolated human NK cells and its expression is only upregulated in a small subpopulation after IL-2 stimulation (158). In that study, it was also shown that DPPIV inhibition suppresses DNA synthesis and cell cycle progression of NK cells, but these effects may be DPP8/9 mediated as the inhibitors used in that study are now known to also inhibit DPP8/9 activity (159). Another study shows that DPPIV is actually only expressed by a small subpopulation of peripheral NK cells (160). The natural cytotoxicity of NK cells is not influenced by the presence or absence of DPPIV on their cell surface (158, 160). However, DPPIV-negative NK cells show significantly less CD16-dependent lysis than DPPIV-positive NK cells (160). Interestingly, NK cytolytic function against tumor cells was diminished in DPPIV-deficient rats in a model for lung metastasis (161).

**Figure 3:**
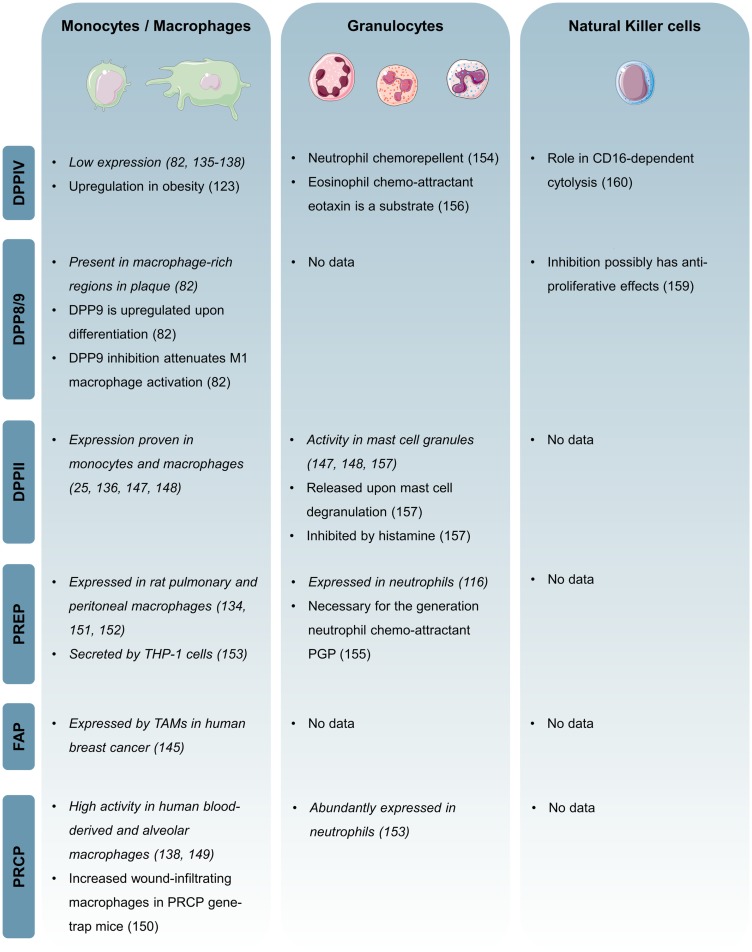
**Overview of the expression and function of individual DPP family members in the innate immune system**. Expression-based evidence is in italic.

Figure 3 shows an overview of published data on the DPP family in the innate immune system.

### The DPP family in the adaptive immune response

#### DPP Family Members in Humoral Immunity

Only about 5% of freshly isolated CD20-positive B cells express DPPIV, but this fraction grows significantly upon pokeweed mitogen (PWM) or *S. aureus* protein stimulation (162). Similar to NK cells, DPPIV inhibitors significantly suppress DNA synthesis in B-lymphocytes (162), but again these inhibitors are now known to also inhibit DPP8 and 9 (159). Mouse spleen-derived B-lymphocytes only express low amounts of DPPIV mRNA (163). DPP8 and 9 mRNA, on the other hand, are expressed at much greater levels in these cells, and they are upregulated in Raji cells, a B-lymphocyte-like cell line, after PWM, LPS stimulation or mitomycin c treatment, and downregulated after DTT treatment (163). DPP8 and 9 have also been shown immunohistochemically in human lymph follicular lymphocytes (164). DPPII activity has also been shown in human B-lymphocytes (25).

#### DPP Family Members in Cell-Mediated Immunity

Dipeptidyl peptidase IV was originally described as a surface marker for T-lymphocytes, in which case it is better known as CD26, and later more specifically for a subset of CD4-positive memory cells, CD4^+^ CD45RO^+^ CD29^+^ cells, which respond maximally to recall antigen tetanus toxoid and induce B-cell IgG synthesis (165, 166). Indeed, CD26 surface expression is augmented along with the antigen sensitivity of a particular CD4^+^ T-cell clone (167). CD26^high^ CD8^+^ T-cells belong to the early effector memory T-cell subset (168). CD26 is also a marker for T-cell activation (165, 169–171). CD26 expression on CD4^+^ T-cells correlates with T_H_1 responses. Stimuli that typically induce a T_H_1 phenotype tend to induce CD26 expression (172). Additionally, the CD4^+^ T cells capable of transendothelial migration *in vitro* are characterized by a bright expression of CD26 (173, 174), but CD26 does not seem to be actually involved in T-cell adhesion to endothelial cells or fibroblasts (175). Recently, it was shown that up to 98% of all T_H_17 cells show very high CD26 expression, with mean fluorescent intensity on these cells almost twice as high as on T_H_1 or T_H_2 cells. Therefore, the authors of this study suggest CD26 as a marker for T_H_17 cells (176). Conversely, CD26 has been proposed as a negative marker for the selection T_reg_ cells due to its very low-surface expression on these cells (176–178).

CD26 is also a costimulatory molecule for T-cell activation. Crosslinking of CD26, along with CD3, stimulates T-cell activation and proliferation (168, 179, 180). CD26 can also directly activate T-cells in an alternative activation pathway, but this requires the presence of the TCR/CD3 complex (181–183). During costimulation, CD26 is mannose-6 phosphorylated and internalized, the latter of which is mediated in part by its interaction with M6P/IGFIIR (184). It then localizes to lipid rafts where it might interact with CD45, required for TCR signaling, facilitating co-localization of this molecule with TCR signaling molecules (185, 186). A number of candidate binding partners for costimulation have been proposed. ADA and CD26 are known binding partners (187). Even though ADA binding to CD26 does not seem to be essential for immune functions in humans (188), the nanomolar affinity of this interaction probably reflects its importance (189). Indeed, association with free ADA or ADA presented by ADA-anchoring proteins on dendritic cells seems to costimulate T-cells through CD26 binding (190, 191). On the other hand, it has been shown that soluble DPPIV enhances T-cell proliferation independent of its enzyme activity or ADA-binding capability (192). Interestingly, the ADA–CD26 interaction can be inhibited by HIV-1 external envelope protein gp120 and this requires interaction of gp120 with CXCR4 (189). In fact, evidence suggests a physical association between CXCR4 and CD26 on peripheral blood lymphocytes (193). Fibronectin is another known binding partner of CD26 involved in T-cell costimulation (194–196). Finally, CD26 interacts with caveolin-1 on monocytes. This interaction causes an upregulation of CD86 on these cells, which potentiates antigen-specific T-cell activation (197). Most studies seem to find no need for DPPIV’s enzymatic activity for succesful costimulation, as evidenced through the use of inhibitors and catalytically impaired DPPIV mutants (198–201).

Dipeptidyl peptidase 8 and 9 are present in baboon spleen interfollicular T-lymphocytes and Jurkat T cells (164). They are upregulated in the latter after PWM and LPS, but not PHA, stimulation (163, 202, 203). Activation of PWM-stimulated T-cells is suppressed after DPPIV/8/9 inhibition. Moreover, DNA synthesis and T-cell proliferation are reduced, as well as production of IL-2, -10, -12, and IFN-γ. This is due to an induction of TGF-β secretion (159, 204–207). Inhibition also upregulates CTLA-4 and downregulates DPPIV expression (206, 208). These observations might be physiologically relevant as endogenous inhibitors of DPPs are known which have similar effects in cell-based experiments as the synthetic inhibitors (209, 210).

Dipeptidyl peptidase II activity is higher in T-lymphocytes than in B-lymphocytes (25) and absence of DPPII steers T-lymphocytes toward a T_H_17 phenotype. T-lymphocytes of DPPII KO mice hyperproliferate and secrete IL-17 after CD3 crosslinking or after *in vivo* priming and *in vitro* antigen-specific restimulation (211). PREP activity has also been shown in mouse T-lymphocytes (212). Its activity is significantly higher in immature, double-positive thymocytes compared to mature, single-positive thymocytes, or peripheral T-cells. T-cells stimulated with Con A followed by IL-2 show a time-dependent increase in PREP activity and pre-treatment of cells with a PREP inhibitor renders them resistant to activation-induced cell death (212).

**Figure 4:**
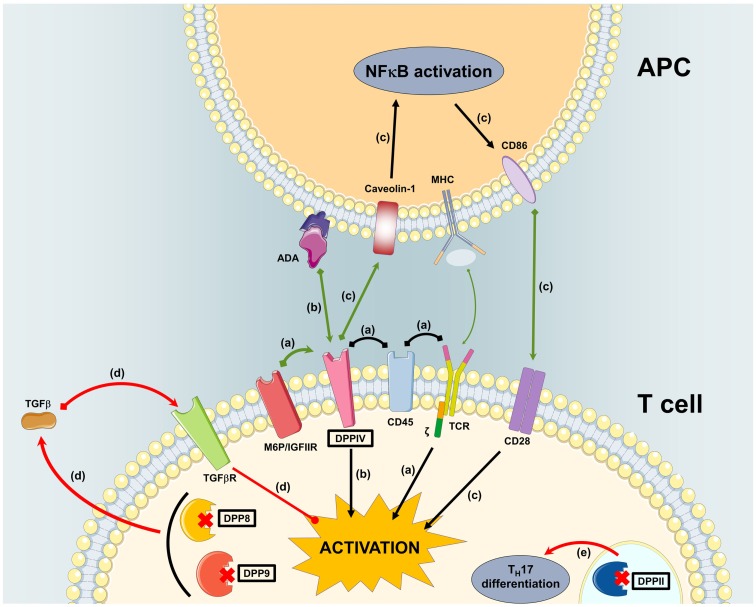
**Overview of *in vitro* data on DPP involvement in primary human T cell activation**. **(A)** M6P/IGFIIR associates with mannose-6-phosphorylated DPPIV causing it to associate with CD45 in lipid rafts. This facilitates co-localization with the TCR signaling molecules for T cell costimulation. **(B)** Interaction of ADA presented by ADA-anchoring proteins on dendritic cells with DPPIV on T cells causes costimulation. **(C)** Interaction of DPPIV on T cells with caveolin-1 on monocytes induces the expression of CD86 on the latter. Interaction of CD86 with CD28 costimulates T cells. **(D)** Inhibition of DPP8/9 induces TGFβ in PWM-stimulated T cells. TGFβ attenuates T cell activation. **(E)** Inhibition or absence of DPPII steers T cells toward T_H_17 differentiation.

Figure 4 shows an overview of *in vitro* data on DPP involvement in primary human T cell activation.

## The DPP Family in Inflammatory Disease

The DPP family has been reported to be dysregulated or even involved in a number of inflammatory disorders. Expression levels of a number of family members are modulated in rheumatoid arthritis. Whereas the density of CD26 on peripheral T cells is increased in patients, it is low on synovial fluid T cells (213–215). DPPIV activity in plasma, serum, or synovial fluid of patients has also been found to be decreased, similar to results in several rat models of arthritis (216–222). Interestingly, rats resistant to induction of arthritis show higher plasma DPPIV levels (222). By contrast, DPPII and PREP activity are increased in serum or synovial fluid of arthritis patients (219–221). Likewise, FAP immunoreactivity is much higher in fibroblast-like synoviocytes of rheumatoid arthritis patients compared to osteoarthritis controls (223). DPPIV’s involvement in rheumatoid arthritis has been studied, but remains unclear. On the one hand, inhibition can suppress development of arthritis in rats (224). Note, however, that effects mediated through other DPPs are hard to exclude as these inhibitors were developed before DPP8 and DPP9 were discovered. On the other hand, induced arthritis is more severe in DPPIV-deficient mice (216). This may be due to increased levels of circulating CXCL12 (216), a DPPIV substrate shown to be involved in rheumatoid arthritis. Several case reports in patients seem to suggest a link between the development of rheumatoid arthritis and the use of DPPIV inhibitors (225–227). PRCP has also been associated with rheumatoid arthritis as its activity was shown in synovial fluid isolated from arthritic joints (149).

Inflammatory bowel disease shows a distinct expression pattern of the DPP family. DPPIV serum or plasma activity seems to be lower in patients, whereas there is an increase of circulating CD26^+^CD25^+^ cells with a higher CD26 surface expression (228, 229). FAP is heavily expressed by myofibroblasts in the submucosa strictures in Crohn’s disease, and is upregulated after stimulation with TNFα or TGFβ (230). In a mouse model, colonic DPPII and DPP8 mRNA and DPPII activity are increased, while colonic DPP8/9 activity only increases significantly in mice that are also DPPIV knockouts (231). In mouse models, inhibition or abrogation of DPPIV seems to at least partially ameliorate symptoms, possibly by increasing circulating GLP-2, impairing neutrophil recruitment, and maintaining T_reg_ populations (231–236). Some of those beneficial effects may be mediated in part by the other DPPs, as additive effects were found for DPPIV KO and the DPP inhibitors (231, 234, 237). A recent study suggests that the ameliorative effects of DPP inhibitors are most likely not mediated through GLP-2 protection (238).

The DPP family has also been studied in neuroinflammation. Ischemia-induced neuroinflammation in rats prompts a distinct expression and activity pattern of the DPPs. In the days following ischemia, the brain of these rats undergoes a complex reorganization of DPP expression with changes in mRNA, protein, and activity levels of DPPII, 4, 8, and 9 in cortical neurons, microglia, and macrophages (146). Similarly, PREP seems to be associated with astrocytes and microglia in lesioned inflamed brains of rats (239). DPPIV and PREP also may be involved in multiple sclerosis. CD26^+^ T cells were found to correlate with disease scores (240). Soluble DPPIV levels are elevated in cerebrospinal fluid of patients (241). Plasma PREP activity, on the other hand, is lower in patients with relapsing–remitting or primary progressive multiple sclerosis and in clinically isolated syndrome (118, 119). Interestingly, PREP inhibition seems to aggravate symptoms in a mouse model of multiple sclerosis (118).

In systemic lupus erythematosus, DPPs also seem to be dysregulated. In mouse models, DPPII and PREP activities are increased in plasma, spleen, kidney, and liver, whereas DPPIV activity is decreased (221, 242). Human patients also show elevated DPPII and reduced DPPIV activities in serum, along with reduced numbers of CD26^+^ T cells (221, 243). Interestingly, serum DPPIV levels are inversely correlated with disease score (243). FAP immunoreactivity is decreased in the synovium of lupus patients (244).

Finally, DPPIV has been studied in psoriasis, an immune-mediated chronic inflammatory disorder with primary involvement of skin and joints. Its mRNA, protein levels and activity are higher in psoriatic skin samples (245, 246). By contrast, serum DPPIV levels and activity seem to be lower in patients (247, 248), accompanied by a reduction of peripheral CD8^+^CD26^+^ T cells (249, 250). Two case reports suggest a link between the use of DPPIV inhibitor sitagliptin and psoriasis. While one woman developed a psoriaform eruption 6 days after starting sitagliptin treatment (251), another patient’s psoriatic lesions gradually diminished and were effectively gone 3 months after the start of sitagliptin treatment (252).

## The DPP Family in Atherosclerosis

Dipeptidyl peptidase IV has recently received much attention for its potential as a therapeutic target for the treatment of atherosclerosis (Box 3) (253). This is not surprising considering the current use of DPPIV inhibitors in the treatment of T2D and the fact that T2D is associated with a higher risk for atherosclerosis (28, 254). In the ApoE^−/−^ mouse model of atherosclerosis, DPPIV inhibition generally reduces plaque area and monocyte and macrophage plaque infiltration (255–257). A reduction in the number of plaque lesions or in smooth muscle cell content have also been observed (255, 256), as well as lower plaque MMP9 and higher plaque collagen levels, suggesting increased plaque stability (258). One study reported effects of DPPIV inhibition on atherosclerotic plaques of only diabetic ApoeE^−/−^ mice (141), but more recently, Terasaki et al. found similar effects in non-diabetic and diabetic ApoE^−/−^ mice (259). Likely, such differences can be explained by the fact that different DPPIV inhibitors were employed. Effects of DPPIV on atherogenesis similar to those observed in ApoE^−/−^ mice have been reproduced in LDLR^−/−^ mice (142, 260). In human atherosclerotic plaques, DPPIV immunoreactivity could only be found on endothelium of neovessels (82). It was recently found that DPPIV activity may be a predictor for the onset of atherosclerosis in otherwise healthy Chinese individuals (261). Another prospective study investigated the influence of vildagliptin or sitagliptin treatment on intima-media thickness, a surrogate marker for atherosclerosis. This study found that treatment with vildagliptin or sitagliptin reduced intima-media thickness, suggesting that DPPIV inhibition might be beneficial in atherosclerosis in humans as well (262). Moreover, treatment naïve T2D patients treated with alogliptin for 3 months saw a significant decrease in their circulating atherogenic lipids (263).

Box 3 **Atherosclerosis.**

Atherosclerosis is the most common underlying cause of cardiovascular diseases and should be regarded as an inflammatory disease. It starts with dysfunction of the endothelium leading to the expression of leukocyte adhesion molecules, such as selectins and integrins. Locally produced pro-inflammatory cytokines attract the immune cells into the inner layer of the endothelium. However, not only leukocytes are found in the plaque but also low-density lipoprotein particles (LDL) and their oxidized counterparts (oxLDL). In the plaque, monocytes differentiate into macrophages, phagocytose the oxLDL and turn into so-called pro-atherogenic foam cells. This process leads to a self-sustaining, local inflammation leading to plaque growth, and migration of smooth muscle cells into the core. A plaque is defined as stable as long as it is contained by a thick fibrous cap. However, the latter is slowly degraded by the proteolytic enzymes from the leukocytes. This eventually leads to rupture and the formation of arterial thrombi (264, 265).

It has been suggested that DPPIV inhibitors’ anti-atherogenic effects are mainly mediated through decreased monocyte infiltration, as DPPIV inhibitors suppress monocyte activation and chemotaxis *in vitro* (142, 258). DPPIV inhibition also reduces *in vitro* foam cell formation in exudate peritoneal macrophages from ApoE^−/−^ mice (255). Moreover, soluble DPPIV stimulates *in vitro* proliferation of smooth muscle cells and this can be reduced through the addition of a DPPIV inhibitor (256, 260). Finally, active circulating GLP-1 levels are augmented and this improves endothelial dysfunction (259, 266). Probably, DPPIV inhibition improves atherosclerosis through a combination of all these mechanisms. Indeed, incretin antagonists only partially attenuate the anti-atherogenic effects of DPPIV inhibition, suggesting that other mechanisms beyond incretin preservation are in play (259). Interestingly, monocyte–endothelial cell adhesion is abrogated by an anti-SDF-1α antibody *in vitro* (267). LDL seems to induce SDF-1α expression and leads to smooth muscle cell proliferation and inhibition of cell apoptosis (267, 268). SDF-1α is a DPPIV substrate, which loses its biological activity after cleavage (216). As DPPIV inhibition seems to improve atherosclerosis, whereas intact SDF-1α appears to be deletorious, it could be argued that SDF-1α cleavage by DPPIV does not play a major role in atherosclerosis.

Dipeptidyl peptidase 8 and 9 have been found to be abundantly present in the macrophage-rich regions of human atherosclerotic plaques and considering DPP9’s role in macrophage activation, it might potentially be involved in atherogenesis (82). FAP expression is enhanced in some, but not all types of human atheromata. It is found on smooth muscle cells, and its expression correlates with macrophage burden, probably due to the fact that TNFα upregulates FAP in smooth muscle cells *in vitro*. As it is mainly associated with collagen-poor regions and can digest type I collagen and gelatin *in vitro*, FAP probably contributes to plaque instability (269).

**Figure 5:**
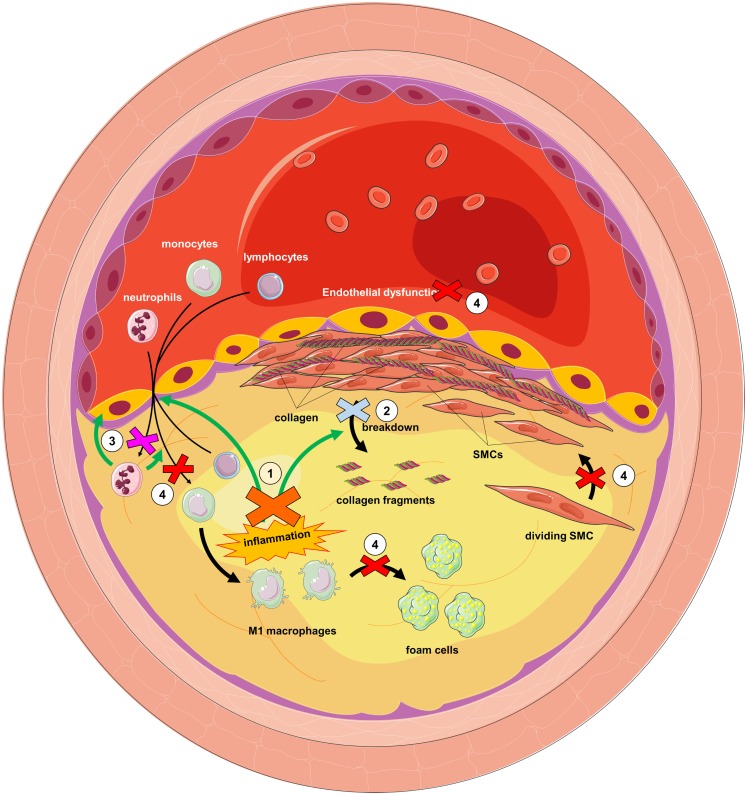
**Dipeptidyl peptidase inhibition as a putative strategy for the treatment of atherosclerosis**. (1) DPP9 inhibition would attenuate M1 macrophage activation, reducing local inflammation. Reduction in TNFα would reduce FAP on smooth muscle cells (SMCs). This and FAP inhibition (2) would reduce collagen degradation and therefore plaque instability. PREP inhibition (3) would reduce neutrophil infiltration and consequently endothelial dysfunction and further monocyte infiltration. DPPIV inhibition (4) would prevent SMC proliferation, foam cell formation, endothelial dysfunction, and monocyte infiltration.

Interestingly, many of the studies reviewed above show the potential of targeting DPP family members for the treatment of atherosclerosis (see Figure 5). FAP inhibition might reduce plaque instability by decreasing collagen breakdown; DPP9 inhibition is likely to attenuate M1 macrophage activation, reducing the local inflammatory cascade; DPPIV inhibition may decrease monocyte infiltration, foam cell formation, improve endothelial dysfunction, and reduce smooth muscle cell proliferation; and finally, PREP inhibition might reduce neutrophil infiltration, preventing endothelial dysfunction, and monocyte infiltration. All of this shows the possibilities of repositioning DPPIV inhibitors, currently being used to treat type 2 diabetes, as well as the potential of targeting other members of the DPP family.

## Conclusion

Caution should be taken when interpreting results from literature data based on DPP inhibitors, especially from older studies. It is now known that, under the experimental conditions used, many of these inhibitors are not specific for one particular family member. The reported findings, however, remain interesting. This review has shown extensive involvement of members of the DPP family in the immune system. It is clear that these enzymes hold great potential as targets for the treatment of certain inflammatory disorders. Particularly, the possibility of targeting DPP family members for the prevention and treatment of atherosclerosis warrants further investigation.

## Conflict of Interest Statement

The authors declare that the research was conducted in the absence of any commercial or financial relationships that could be construed as a potential conflict of interest.
